# Risk factors of developmental dysplasia of the hip in a single clinical center

**DOI:** 10.1038/s41598-022-24025-8

**Published:** 2022-11-14

**Authors:** Huan Xiao, Yi Tang, Yuxi Su

**Affiliations:** 1 Department of Ultrasound, Chongqing Key Laboratory of Pediatrics, Ministry of Education Key Laboratory of Child Development and Disorders, National Clinical Research Center for Child Health and Disorders, China International Science and Technology Cooperation Base of Child Development and Critical Disorders, Children’s Hospital of Chongqing Medical University, Chongqing, People’s Republic of China; 2 Department of Orthopaedics, Chongqing Key Laboratory of Pediatrics, Ministry of Education Key Laboratory of Child Development and Disorders, National Clinical Research Center for Child Health and Disorders, China International Science and Technology Cooperation Base of Child Development and Critical Disorders, Children’s Hospital of Chongqing Medical University, Yuzhong District Zhongshan 2 Road 136#, Chongqing, 400014 People’s Republic of China

**Keywords:** Risk factors, Epidemiology, Paediatric research

## Abstract

Developmental dysplasia of the hip (DDH) is the main cause of early-onset hip osteoarthritis in adulthood. Early screening of DDH is the key to avoiding these severe complications. This study aimed to assure the risk factors are suitable for screening patients with DDH in our region. We retrospectively analyzed 10,668 patients (21,336 hips) at our hospital. Overall, 204 patients with pathological DDH and 408 patients with normal hips were included in this study. All patients were diagnosed by performing ultrasound examinations according to the Graf technique. The risk factors were assessed based on patients’ clinical data. Pearson’s chi-square or Fisher’s exact tests and multivariate logistic regression analysis were performed for statistical analysis. A total of 204 patients were diagnosed with pathologic DDH and were treated with the Pavlik harness. Among these, 184 patients were female. There were 73 cases of first birth, 13 had oligohydramnios, 13 had foot deformity, 31 had breech delivery, 6 had congenital muscular torticollis. Female sex, vaginal delivery, breech presentation, oligohydramnios and foot deformity were identified as the risk factors for DDH. The risk factors of DDH in our clinical center were confirmed in our clinical center, this can supply the screening advice for the doctors.

## Introduction

Ultrasound (US) examination for the diagnosis of developmental dysplasia of the hip (DDH) in children aged < 6 months is widely accepted worldwide^1–3^. Countries including Austria, Sweden, Germany, the Czech Republic, and Switzerland introduced the national screening of neonatal hips using the Graf technique^4^. In other countries, such as England, the USA^5^, and Turkey^6,7^, children with risk factors detected during physical examinations are referred to specialists for further diagnosis using US examinations^6,8^. Since risk factors vary from one country and area to another, it is important for each country or local area to establish their own screening standards^6,9,10^. Our region, located in the southeast of China, is densely populated, there is also necessary for establishing our own risk factors. In this retrospective study, we aimed to analyze the data of patients diagnosed with DDH treated with the Pavlik harness, a brace or a cast, or open surgery and establish an effective screening system, using a combination of risk factor assessment and US examination, that is suitable for screening patients with DDH in our region.

## Materials and methods

### Patients and methods

**Figure 1:**
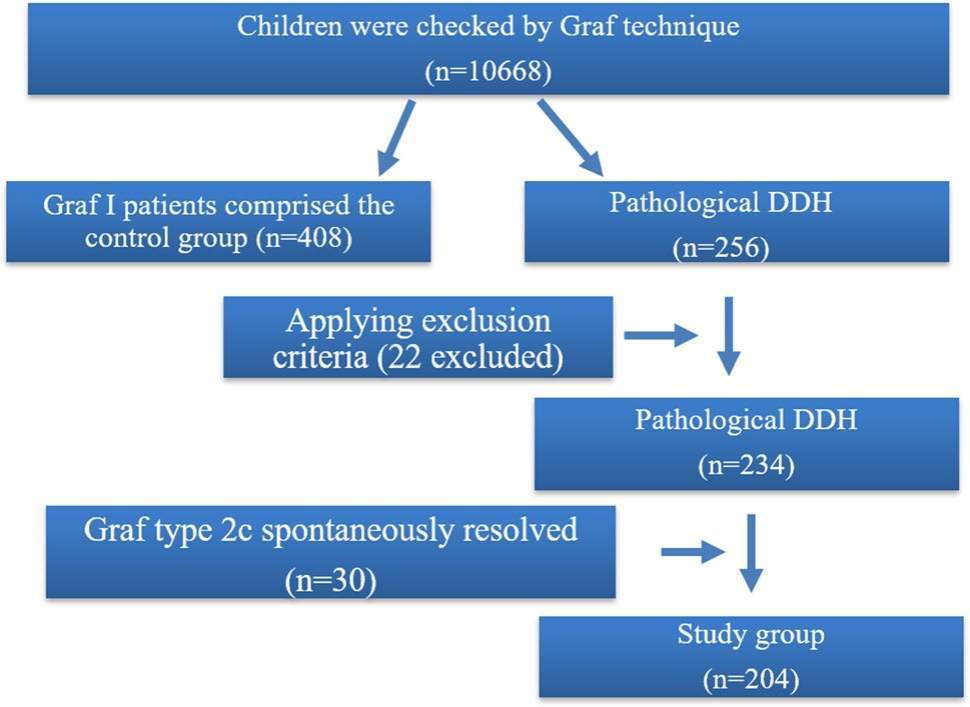
Schematic diagram showing the study design. DDH, developmental dysplasia of the hip.

**Figure 2:**
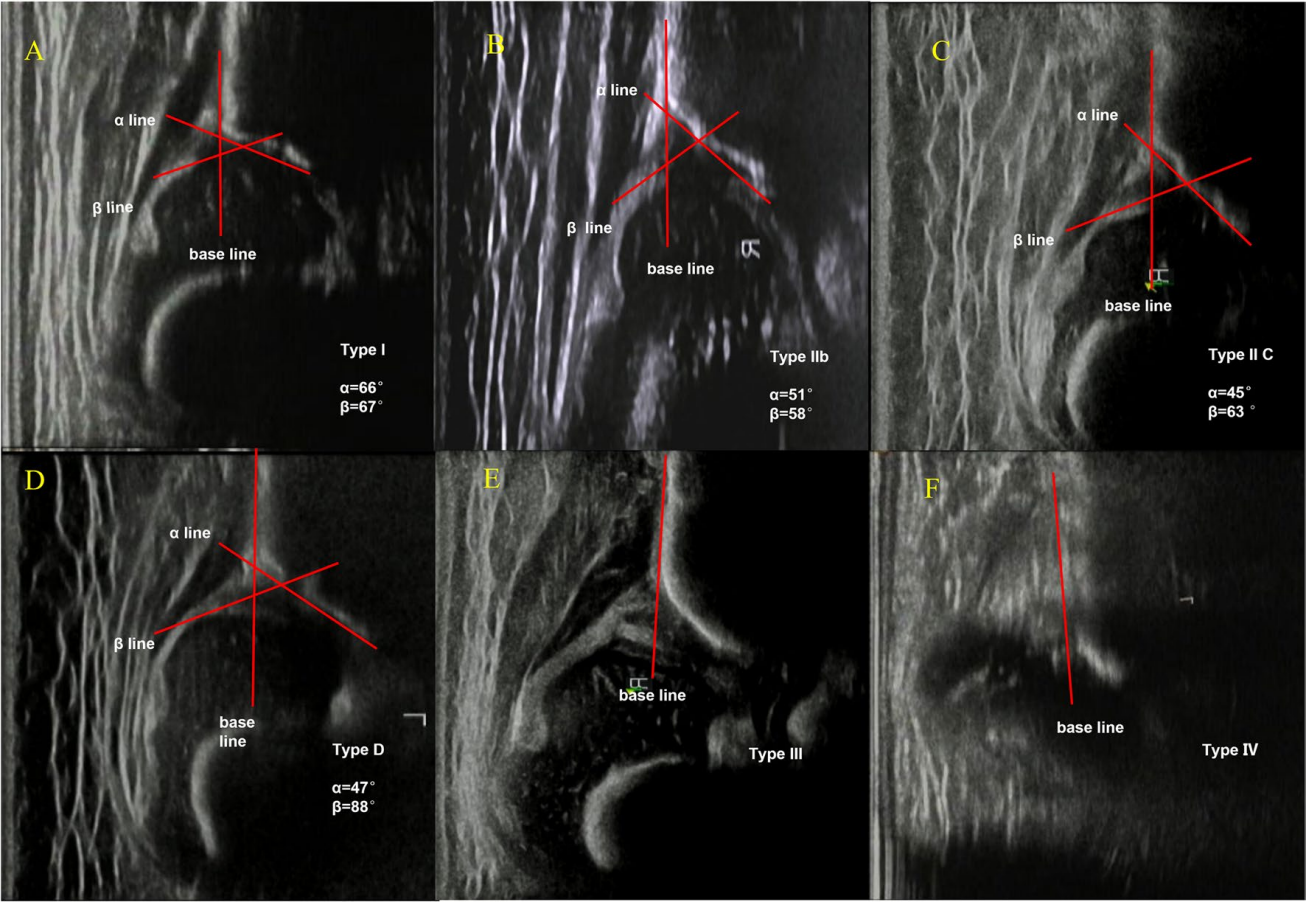
Graf technique for classifying different types of developmental dysplasia of the hip. A shows the Graf type technique for a 2-month-old female infant. B shows the Graf type IIb technique for a 3-month-old female infant. C shows the Graf type IIc technique for a 2-month-old female infant. D shows the Graf type D technique for a 3-month-old female infant. E shows the Graf type III technique for a 3-month-old female infant. F shows Graf type IV technique for a 3-month-old female infant.

This single-center retrospective study conducted between January 2014 and December 2018 analyzed the clinical data of 10,668 patients (Tables 1and 2). All patients had undergone US examination according to the Graf technique. Patients were included if their condition was diagnosed as true DDH (Fig. 1). Among those diagnosed with DDH, we further categorized the patients into the following two subgroups: patients with acetabular dysplasia and those with dislocated or subluxated hips. Acetabular dysplasia is not clinically manifested and is typically categorized as Graf type II by ultrasonography. Children aged < 3 months categorized as Graf type II patients were followed-up until the 3rd month of life. If the condition did not resolve by the 3rd month, they were included in our study and classified as having acetabular dysplasia. The other group comprised patients with dislocated or subluxated hips were classified as Graf type III/IV patients by ultrasonography. Infants aged > 3 months with abnormal US findings (Graf classification types IIc, D, and III/IV) comprised the case group. Patients were excluded if they had teratologic dislocations or neuromuscular diseases, which affect the peripheral nerves, neuromuscular junctions, and muscle tissue and mainly manifest as decreased or lost muscle contractility and muscle atrophy, or if clinical data was incomplete. Physical examination for DDH was conducted for each infant when they visited the local health doctor. Patients with abnormal presentation were referred to our hospital. In our hospital, detailed physical examinations were conducted, and the risk factors were recorded in the patients’ clinical data. The Graf method, which was developed by Graf, who used B-ultrasound for the early diagnosis of DDH and established a quantitative typing system in 1980, was performed for all patients, and based on the patients’ Graf classification, the Pavlik harness was used if needed. The sonographic examination for each child was performed by the same team, who were trained and certified by the National Graf Technique Training Plan (Fig. 2). The Hitachi Aloka Color Ultrasound machine (Erang God HI VISION Preirus) was used to perform the US examination according to the Graf method. The US examinations were performed by four doctors with 5–8 years of experience. Children were asked to wear a close-fitting coat with no diapers and were positioned sideways in a special small bed, with the patient’s leg inside the bed. During this process, their mothers ensured that they were calm, the doctors held the probe parallel to the inspection before and after the side of bed, and the image was frozen and measured until the standard section appeared.

**Table 1:** Risk factors for the screening of developmental dysplasia of the hip.

Risk factors	DDH group (n = 204)		Control group (n = 408)		p value	OR (95% CI)	RR (95% CI)
	Positive	Negative	Positive	Negative			
Sex (female, positive)	184	20	194	214	< 0.0001	10.15 (6.274–16.66)	5.695 (3.743–8.794)
Breech delivery	31	173	4	404	< 0.0001	0.1306 (0.08496–0.2007)	0.2315 (0.1636–0.3277)
Vaginal delivery	53	151	184	224	< 0.0001	0.2558 (0.1768–0.3702)	0.3923 (0.2995–0.5138)
First birth	73	131	172	236	0.1293	1.178 (0.9545–1.470)	0.8488 (0.6804–1.048)
Oligohydramnios	13	191	3	405	< 0.0001	9.188 (2.587–32.63)	2.535 (1.949–3.298)
Birth weight	3.21	3.11	2.98	3.05	0.5506		
Congenital muscular torticollis	6	198	1	407	0.0066	12.33 (1.474–103.2)	2.619 (1.895–3.619)
Foot deformities	13	191	2	406	< 0.0001	13.83 (3.589–61.89)	13.00 (3.316–51.21)
Family history	3	201	1	407	0.1104	6.075 (0.6276–58.80)	2.269 (1.274–4.040)
Swaddling	9	195	27	381	0.3623	0.6513 (0.3003–1.412)	0.7385 (0.4146–1.315)

*OR* odds ratio, *RR* relative risk, *CI* confidence interval, *DDH* developmental dysplasia of the hip.

p < 0.05 indicates a significant difference.

**Table 2:** Risk factors for the screening of DDH by binary logistic regression analysis.

Risk Factors	OR (95% CI)	p value*
**Sex**		
Female	1	< 0.001
Male	0.024–0.101	
**Oligohydramnios**		
Normal	1	0.004
Decreased	0.011–0.417	
**Vaginal delivery**		
Yes	1	0.014
No	1.116–2.604	
**Breech delivery**		
No	1	< 0.001
Yes	0.008–0.110	
**Foot deformities**		
No	1	< 0.001
Yes	0.004–0.137	
**Congenital muscular torticollis**		
No	1	0.144
Yes	0.12–1.907	
**Family history**		
No	1	0.253
Yes	0.027–2.594	

*OR* odds ratio, *CI* confidence interval, *DDH* developmental dysplasia of the hip.

*p < 0.05 indicates a significant difference.

A control group was included for the analysis. The control group comprised patients with Graf type I hips, which were considered mature, selected from the inpatients of the neonate, pneumology, hepatology, gastroenterology, and infectious disease departments whose diseases were not related to a congenital deformity. The control group comprised 408 children, which was twice the number of children in the case group.

Our hospital’s ethics committee approved this study. Informed signed consent was obtained from the parents or guardians of the patients. This study is reported in line with the STROCSS (Strengthening the Reporting of Cohort Studies in Surgery) criteria. All methods were performed in accordance with the relevant guidelines and regulations.

### Follow-up

After the patients were diagnosed with DDH at the first visit and the use of the Pavlik harness was prescribed, the patients were followed-up for 2–3 weeks. Based on the results of the Graf analysis that was performed the second time, further treatment, such as the continued use of the Pavlik harness, use of a brace or a cast, or open reduction surgery, was provided. All patients were followed-up for at least 24 months after the final diagnosis.

### Outcome assessments

For the patients aged > 6 months, radiographic parameters were evaluated using the anteroposterior pelvic radiographs. All angle measurements were performed by the same orthopedic surgical team. The acetabular index was measured, and the continuity of Shenton’s line was recorded.

### Statistical analysis

All statistical analyses were performed by using SPSS software version 20.0 (IBM Corp., New York, USA). Continuous variables were analyzed by independent t-tests and categorical variables by χ^2^-tests. Multivariate logistic regression analysis was performed for statistical analysis. Univariate analyses using Pearson’s chi-square and Fisher’s exact tests were performed when the expected count was < 5. A p-value < 0.05 was considered statistically significant.

### Ethics approval and consent to participate

The ethics committee of the Children’s Hospital of Chongqing Medical University approved the study.

### Consent for publish

Informed consent for publication of photographs was obtained from all subjects.

## Results

Graf types III and IV ultrasonographic findings were detected in 125 hips of 110 children, and 54 hips of 47 patients showed ultrasonographic findings of Graf type IId. A total of 91 hips of 70 patients showed Graf type IIc findings; however, only 47 patients were included in this study, because the pathological condition of the other 30 patients spontaneously resolved without treatment (Table 3). A total of 204 patients were diagnosed with pathologic DDH and treated with the Pavlik harness. Among these, 184 were female, 31 were breech births, 53 were vaginally delivered, 73 were first births, 13 had oligohydramnios, 13 had foot deformities, 6 had congenital muscular torticollis, 137 had limited hip abduction, 23 had Ortolani and Barlow maneuver positivity, and 9 were swaddled, with an average birth weight of 3.24 kg. In total, 408 patients were included in this study as the control group, and all patients were diagnosed to have normal hips (Graf type I). Among these, 44 were female, 4 were breech births, 184 were vaginally delivered, 172 were first births, 3 had oligohydramnios, 2 had foot deformities, 1 had congenital muscular torticollis, 1 had a family history of DDH, 27 were swaddled, and none had Ortolani and Barlow maneuver positivity, with a mean birth weight of 2.98 kg. Compared with the control group, female sex, breech delivery, vaginal delivery, oligohydramnios, foot deformities were identified as the risk factors for DDH in the case group (Tables 1 and 2). In our study, 15.20% (31/204) and 0.99% (4/404) of the patients in the case and control groups, respectively, had breech presentation, which showed a significant difference between the two groups (p < 0.0001). Thirteen children with DDH and only two children in the control group presented with a foot deformity, and the difference was significant (p < 0.0001) (Tables 2 and 4). The average acetabular index and continuity of Shenton 's line was shown in Table 3.

**Table 3:** Screening results according to the Graf technique.

Graf technique	Screened patients	Included in this study	AI (degree)	Continuity of Shenton 's line
Type I	9719	408	27.5 ± 2.7	408
Type IIa	533	0	N/A	N/A
Type IIb	155	0	N/A	N/A
Type IIc	77	47	32.7 ± 8.0	39
Type D	57	47	32.1 ± 6.1	0
Type III	89	77	37.5 ± 6.9	0
Type IV	38	33	36.0 ± 7.3	0

*AI* acetabular index.

**Table 4:** Detailed description of the foot deformity.

Patients	Sex	Graf type		Foot deformity	
		Left	Right	Left	Right
1	M	IV	IV	CTEV	CTEV
2	M	I	IV	N	CTEV
3	M	IV	IV	CTEV	CTEV
4	M	IIIa	IIIa	CTEV	CTEV
5	F	IV	IV	CTEV	N
6	F	IV	IV	CV	CV
7	F	IIIa	IIIa	CTEV	CTEV
8	F	I	IIIa	CV	N
9	F	IV	IV	CTEV	CTEV
10	F	IV	I	CTEV	N
11	F	IV	IV	CTEV	CTEV
12	F	IIIa	IIa	CV	CTEV
13	F	IIIa	IIc	CTEV	CTEV

*M* male, *F* female, *N* normal, *CTEV* congenital talipes equinovarus, *CV* calcaneous valgus.

## Discussion

Recently, screening of DDH in young children has become widely accepted^4,11^. Early treatment of DDH could provide better results compared to late treatment. Considering that China is a developing country, a new screening system, which is suitable for our region, should be established. Currently there are three kinds of screening systems, which are as follows: clinical examination alone, general screening using US, and a combination of US and risk factor screening^4^. The American Academy of Pediatrics recommends that US should be used to evaluate female infants born via breech delivery and those with a family history of DDH in the USA^12^. In the UK, a family history of DDH, breech position, multiple births, and clinical hip instability were the indicators for US examination for the diagnosis of DDH. In this study, we also attempted to adopt the methods used in the USA and UK^13^. There are two limitations associated with the use of only US screening in our region. Although the US technique is affordable, costing approximately 20 US dollars, the procedure must be performed by an experienced ultrasonographer. The second major reason for not adopting full screening was that US machines were not readily available in some rural areas in our region. In this study, we successfully established our own suitable screening method that combined risk factor assessment and US examination, which effectively increases the screening efficiency and decreases the cost of DDH in our region.

Several risk factors are associated with DDH^8,14^. Some studies reported a strong correlation between DDH and a family history of DDH, breech presentation, swaddling, and congenital foot deformity^8,11,15,16^. In this study, we aimed to confirm the correlation between DDH and the risk factors of DDH in our region. We included other risk factors that may also be important, such as diabetes, hyperthyroidism, hypothyroidism, hepatitis B infection, pulmonary tuberculosis infection, and pregnancy-induced hypertension, during the pregnancy period. In our country, vaccinations are scheduled at the ages of 1, 3, 4, and 5 months. Doctors are advised to examine the hip during these visits. We also analyzed the number of pregnancies, number of gestational weeks, gestational age, oligohydramnios, delivery method (cesarean section or vaginal delivery), breech presentation, birth weight, foot deformity, congenital torticollis, swaddling, family history (minimal in the control group), and pregnancy-related diseases (diabetes and hyperthyroidism). The results showed that only female sex, vaginal delivery, breech delivery, foot deformity, oligohydramnios were risk factors for DDH in our area, which contradicts the findings of previous studies^6,11^.

There has been no consensus on the risk factors for congenital muscular torticollis. Breech presentation is considered one of the risk factors for torticollis. This may lead to misleading conclusions regarding congenital muscular torticollis if breech presentation is included in the screening. In our study, 15.20% (31/204) and 0.99% (4/404) of the patients in the case and control groups, respectively, had breech presentation, showing a significant difference (p < 0.0001). We therefore recommend that children with breech presentation should be included in US examinations.

Some studies reported that foot deformities, including congenital talipes equinovarus, may be correlated with DDH^17^. In this study, 13 children with DDH and only 2 children in the control group presented with a foot deformity, and the difference was significant (p < 0.0001).

Breech presentation is considered a risk factor for not only congenital muscular torticollis but also DDH. Many studies have reported a strong correlation between breech presentation and DDH^15,18^. Lambeek et al.^19^ proved that cephalic presentation is associated with a decreased risk of DDH compared to breech presentation, and female children are at a higher risk. In our study, 31 of 204 infants were born via breech delivery (p < 0.0001), and breech delivery was strongly correlated with DDH. Panagiotopoulou et al.^18^ also reported that breech infants delivered via cesarean section presented with a significantly lower risk of DDH; our study showed the same results as others. However, the DDH group showed a lower incidence of first birth, and first birth was not significantly correlated with DDH. There was a government policy in China in 2016 that allowed families to have a second child. Therefore, there were many second-born children within the study period. Further studies involving more patients should be conducted to evaluate this.

The factors related to limited fetal mobility, such as oligohydramnios and high birth weight, presented an increased risk for DDH^20,21^. In our study, we found that oligohydramnios posed an increased risk. In the DDH group, 13 out of the 204 patients presented with oligohydramnios, whereas in the control group, 3 out of 408 infants presented with oligohydramnios (p < 0.0001). However, there was no significant difference in the proportion of infants with high birth weight between the two groups (p = 0.5506).

The swaddling method is also considered a risk factor. Most surgeons suggest that parents should not swaddle the infants tightly. This was confirmed by Yamamuro et al.^22^, who launched a national campaign in 1975 to prevent prolonged extension of the hips and knees of infants during the early postnatal period; as a result of this campaign, the incidence of DDH decreased significantly. In our study, no significant difference was found in the use of the swaddling method between the case and control groups. This is because the parents did not adopt the custom of keeping the infants’ hip in prolonged extension, such as that in the swaddling method. The temperature in our region is not low; therefore, tight swaddling is not necessary^23^.

The indications for the early treatment of DDH remain controversial^24^. Some investigators monitor children with type IIa hips and treat those with IIc or D hips, whereas others follow-up patients with type IIc and D hips and treat patients with type III and IV hips^25^. In our study, 77 patients were diagnosed as having type IIc hips; however, the retrospective analysis of the clinical data showed that only 61.0% (47/77) of the patients were treated with the Pavlik harness, and the remaining 30 patients were not cooperative during follow-up or were lost to follow-up; thus, these 30 patients were excluded from our study. There is much controversy regarding the patients who require treatment^26^. Peled et al.^27^ reported that the incidence of suspected DDH requiring treatment is < 0.5%. Based on his definition, 1.91% of our patients required treatment and were diagnosed as having true DDH, which was almost four times higher than that reported in their study. There were some reasons that could have led to the higher rate observed in this study compared to that reported in the study by Peled et al. First, the incidence of DDH was high in our local area. Second, in our study, the first round of patient screening was conducted by doctors, indicating that our clinical screening system may be effective. In general, there was no significant change in our region compared to the other regions.

There are some limitations to this study. First, this was a retrospective study. A prospective study must be conducted to validate our methods and results. Second, the follow-up period was only > 12 months; a longer follow-up duration is, thus, needed, especially for acetabular dysplasia-type DDH cases. Third, our sample size was small; future studies should recruit a larger cohort of patients. Fourth, not all the patients included in this study were neonates; this is a concern because early treatment can produce good results. Fifth, the treatment results were not assessed; this should be prioritized in future studies. Sixth, the control group did not include healthy individuals; therefore, our method may also introduce bias. Finally, the patients with DDH in this study may have included some patients with syndromic entities, heritable bone- or connective tissue disorders, or some other related disease, which could have also introduced bias.

## Conclusions

Our study showed that female sex, vaginal delivery, breech presentation, and oligohydramnios, foot deformities are the risk factors for DDH in our region. The established screening method that combines risk factor assessment and US examination can effectively increase the screening efficiency and decrease the cost associated with the medical care of DDH patients in our region.

## Data Availability

Data of this study can be available if contacted to corresponding author for reasonable request.

## Abbreviations

DDH: Developmental dysplasia of the hip
US: Ultrasound
STROCSS: Strengthening the reporting of cohort studies in surgery
